# Pleiotropic effects of vitamin D_3_ on CD4^+^ T lymphocytes mediated by human periodontal ligament cells and inflammatory environment

**DOI:** 10.1111/jcpe.13283

**Published:** 2020-04-13

**Authors:** Christian Behm, Alice Blufstein, Johannes Gahn, Barbara Kubin, Andreas Moritz, Xiaohui Rausch‐Fan, Oleh Andrukhov

**Affiliations:** ^1^ Division of Conservative Dentistry and Periodontology University Clinic of Dentistry Medical University of Vienna Vienna Austria

**Keywords:** CD4‐Positive T Lymphocytes, co‐culture, Immunomodulation, Periodontal Ligament, Vitamin D

## Abstract

**Aims:**

Both, vitamin D_3_ and human periodontal ligament cells (hPDLCs) possess immunosuppressive properties, but their combined effect on immune cells has never been investigated. Here, we analysed the impact of vitamin D_3_ on the immunosuppressive properties of hPDLCs towards CD4^+^ T lymphocytes.

**Material and Methods:**

Allogenic CD4^+^ T lymphocytes were activated by phytohemagglutinin either in monoculture or co‐culture with hPDLCs, in the presence or absence of IFN‐γ and 1,25(OH)_2_D_3_. After 5 days, CD4^+^ T‐lymphocyte proliferation, CD4^+^ CD25^+^ FoxP3^+^ regulatory T lymphocytes (T_regs_) proportion and IL‐10, TGF‐β1 and IL‐17A production were analysed.

**Results:**

In monoculture, 1,25(OH)_2_D_3_ suppressed CD4^+^ T‐lymphocyte proliferation, increased the percentage of CD4^+^ FoxP3^+^ CD25^+^ FoxP3^+^ T_regs_ and enhanced IL‐10 and TGF‐β1 production. In the presence of IFN‐γ treated hPDLCs, 1,25(OH)_2_D_3_ significantly increased CD4^+^ T‐lymphocyte proliferation and decreased the percentage of CD4^+^ CD25^+^ FoxP3^+^ T_regs_. IL‐10 and IL‐17A expression was significantly diminished by 1,25(OH)_2_D_3_, whereas TGF‐β1 was slightly increased. The effects of 1,25(OH)_2_D_3_ in co‐culture were reversed by inhibition of indoleamine‐2,3‐dioxygenase‐1, prostaglandin‐endoperoxide synthase and programmed cell death 1 ligand 1. 1,25(OH)_2_D_3_ also suppressed the expression of these proteins in hPDLCs.

**Conclusion:**

Effects of vitamin D_3_ on CD4^+^ T lymphocyte are modified by hPDLCs depending on the microenvironment.

**Clinical relevance**

*Scientific rationale for the study:* Although vitamin D_3_ exerts immunosuppressive effects, its role in periodontitis remains controversial. Studies investigating the effect of vitamin D_3_ in complex in vitro co‐culture models are important to clarify its exact role.

*Principal findings:* Vitamin D_3_ suppressed CD4^+^ T‐lymphocyte proliferation and enhanced the proportion of CD4^+^ CD25^+^ FoxP3^+^ T_regs_. However, in the presence of human periodontal ligament cells and inflammatory stimuli, the opposite effects were observed: stimulation of CD4^+^ T‐lymphocyte proliferation and reduction of the CD4^+^ CD25^+^ FoxP3^+^ T_regs_ proportion.

*Practical implications:* The effects of vitamin D_3_ in the periodontium are multifaceted, which should be considered for its application in clinical practice.

## 1 INTRODUCTION

Human periodontal ligament cells (hPDLCs) fulfil the minimal criteria of mesenchymal stem cells (MSCs) (Seo et al., 2004), including the expression of specific surface markers and a multilineage differentiation potential (Viswanathan et al.., 2019). This heterogeneous population of fibroblast‐like cells participates in tissue regeneration by cell proliferation, differentiation into tissue‐specific cells and modulating immune and inflammatory responses (Racz et al., 2014; Wada, Gronthos, & Bartold, 2013; Xiao & Nasu, 2014).

Similarly to other MSCs, hPDLCs possess immunomodulatory properties and affect various immune cells. This is facilitated by the production of enzymes and soluble factors, as well as via direct cell‐to‐cell contact (Andrukhov, Behm, Blufstein, & Rausch‐Fan, 2019; Wada et al., 2013). The key factors mediating these immunomodulatory effects are indoleamine‐2,3‐dioxygenase‐1 (IDO‐1), prostaglandin E_2_ (PGE_2_), programmed cell death 1 ligand 1 (PD‐L1), programmed cell death 1 ligand 2 (PD‐L2) and others (Andrukhov et al., 2019; Wada et al., 2013). Beside their immunosuppressive abilities, hPDLCs can also favour the inflammatory response under certain conditions (Andrukhov et al., 2019), mainly by producing several pro‐inflammatory cytokines, particularly interleukin (IL)‐1β, IL‐6, IL‐8 and monocyte chemoattractant protein 1, which are triggered by different bacterial and viral pathogens (Andrukhov et al., 2016; Behm et al., 2019; Blufstein et al., 2019; Kato, Taguchi, Tominaga, Umeda, & Tanaka, 2014). Among different immune cells, the effects of hPDLCs on CD4^+^ T lymphocytes are the most investigated (Wada et al., 2013). It is known that hPDLCs per se suppress the activation, proliferation and differentiation of CD4^+^ T lymphocytes, enhance the formation of regulatory T lymphocytes (Tregs) and reduce IL‐17A production (Castro‐Manrreza & Montesinos, 2015; Liu, Xu, et al., 2012). These immunosuppressive effects of hPDLCs are strongly enhanced by pro‐inflammatory stimuli such as interferon (IFN)‐γ. Presumably, this is due to increased expression of diverse immunomodulatory proteins, including IDO‐1, prostaglandin‐endoperoxide synthase‐2 (PTGS‐2) and PD‐L1 (Chabannes et al., 2007; English et al., 2009; Meisel et al., 2004; Nasef et al., 2007; Sato et al., 2007; Selmani et al., 2008).

Vitamin D_3_ is a hormone involved in the regulation of bone homoeostasis (Lips, 2006) and immune response (White, 2008). The major sources of vitamin D_3_ are its production in the skin through sun exposure and dietary supplements. Vitamin D_3_ is converted into 25(OH)D_3_ and further to the biologically most active form 1,25(OH)_2_D_3_ mainly in the kidney (Jones, 2008; Lips, 2006). Recent studies show that local conversion of 25(OH)D_3_ into its bioactive form also occurs by immune cells (Wu, Ren, Nguyen, Adams, & Hewison, 2007; Zehnder, 2001) and hPDLCs (Liu, Meng, & Hou, 2012). It is known that 1,25(OH)_2_D_3_ and 25(OH)D_3_ suppress the production of pro‐inflammatory mediators by hPDLCs in response to different stimuli (Andrukhov et al., 2014; Hosokawa, Hosokawa, Shindo, Ozaki, & Matsuo, 2015; Nebel et al., 2015; Tang, Pan, & Zhao, 2013). However, the effect of vitamin D_3_ on the immunosuppressive properties of hPDLCs is unknown.

In the present study, we investigated the effect of vitamin D_3_ on allogenic CD4^+^ T lymphocytes in the presence of hPDLCs and inflammatory stimuli. Particularly, we examined the influence of IFN‐γ treated hPDLCs on the potential of 1,25(OH)_2_D_3_ to affect CD4^+^ T‐lymphocyte proliferation, CD4^+^ CD25^+^ FoxP3^+^ T_regs_ proportion and production of functional cytokines by CD4^+^ T lymphocytes in an indirect in vitro co‐culture model. We additionally inhibited IDO‐1, PD‐L1 and PTGS‐2 proteins pharmacologically in order to evaluate their contribution into hPDLCs’ mediated effects on CD4^+^ T lymphocytes. Further, we investigated the effect of 1,25(OH)_2_D_3_ on the production of IDO‐1, PTGS‐2, PD‐L1 and PD‐L2 by IFN‐γ treated hPDLCs in vitro.

## 2 MATERIAL AND METHODS

Detailed cell isolation and analysis protocols can be found in the Supporting information.

The study protocol was approved by the Ethics Committee of the Medical University of Vienna (EK Nr. 1694/2015, extended 2019). All procedures were performed according to the Declaration of Helsinki and the Good Scientific Practice Guidelines of the Medical University of Vienna.

### 2.1 Co‐culture of hPDLC and CD4^+^ T lymphocytes

2.5 × 10^5^ primary hPDLCs were seeded per well in a 6‐well plate for 24 hr in DMEM supplemented with 10% foetal bovine serum (FBS) and 1% penicillin/streptomycin (P/S). hPDLCs were stimulated with 100 ng/ml IFN‐γ (PeproTech) in the presence or absence of 100 nM 1,25(OH)_2_D_3_ (Cayman Chemical) in FBS‐free DMEM. After 48 hr incubation, the medium was changed to RPMI‐1640 (Sigma‐Aldrich) supplemented with 10% FBS, 1% P/S and 100 ng/ml IFN‐γ and 100 nM 1,25(OH)_2_D_3_. Transwell (TC) inserts with 0.4 µm pores (Sarstedt), containing 1 × 10^6^ freshly isolated allogenic CD4^+^ T lymphocytes, were placed into hPDLC‐containing wells. CD4^+^ T‐lymphocyte proliferation was induced by 10 µg/ml phytohemagglutinin‐L (PHA‐L; ebioscience). In another series of experiments, IDO‐1, PD‐L1 and PTGS‐2 were pharmacologically inhibited to investigate their influence on the effect of 1,25(OH)_2_D_3_ on CD4^+^ T‐lymphocyte proliferation and the functional cytokine production of CD4^+^ T lymphocytes in the presence of hPDLCs. In these experiments, hPDLCs were additionally treated with either 50 µM IDO‐1 inhibitor PF‐06840003, 1 µM PD‐1/PD‐L1 interaction inhibitor BMS202 or 1 µM PTGS‐2 inhibitor Celecoxib (all from Selleck Chemicals) before and during indirect co‐culture. After 5 days of incubation, flow cytometry analysis was performed to measure CD4^+^ T‐lymphocyte proliferation and the proportion of CD4^+^ CD25^+^ FoxP3^+^ T_regs_. Additionally, the levels of IL‐6, IL‐10, IL‐17A and TGF‐β1 were measured by enzyme‐linked immunosorbent assay (ELISA). Solely seeded CD4^+^ T lymphocytes which were stimulated with 100 ng/ml IFN‐γ in the presence and absence of 100 nM 1,25(OH)_2_D_3_ served as reference.

### 2.2 Expression of immunomodulatory proteins

Primary hPDLCs were seeded in 6‐well plates using 2.5 × 10^5^ cells per well in 3 ml DMEM, supplemented with 10% FBS and 1% P/S. After 24 hr, hPDLCs were stimulated with 100 ng/ml IFN‐γ in the absence and presence of different 1,25(OH)_2_D_3_ concentrations (0.01–100 nM) using FBS‐free DMEM containing 1% P/S. Additionally, hPDLCs were stimulated with 100 nM 1,25(OH)_2_D_3_ in the absence of IFN‐γ. Unstimulated cells served as control. After 48 hr, gene expression of IDO‐1, PD‐L1, PD‐L2 and PTGS‐2 were analysed by qPCR. Protein levels were assessed by immunostaining, followed by flow cytometry analysis. IDO‐1 enzymatic activity was analysed by measuring L‐kynurenine concentration.

## 3 RESULTS

### 3.1 Multiparameter analysis of cell surface marker expression in hPDLCs

The stem cell character of hPDLCs was verified by investigating the expression of mesenchymal and hematopoietic stem cell surface markers (Table S1), according to the International Society for Cell and Gene Therapy (Dominici et al., 2006; Viswanathan et al., 2019). Our single‐parameter analysis showed that more than 95% of hPDLCs were positively stained for CD29, CD73, CD90 and CD105. A multiparameter flow cytometry analysis and quadruple‐gating strategy showed that 92.6% of the starting cell population possesses the CD105+/CD29+/CD73+/CD90+/CD31−/CD34−/CD45− full phenotype. This is in line with a previous study showing a decreased percentage of MSC surface marker expression in a multiparameter setting (Chan, Heathman, Coopman, & Hewitt, 2014), compared to single stain analysis. Additionally, a single‐parameter setting showed that about 46% of hPDLCs express CD146, which is in line with a previous study showing about 43% CD146^+^ cells (Zhu et al., 2013).

### 3.2 CD4^+^ T‐lymphocyte proliferation

**Figure 1:**
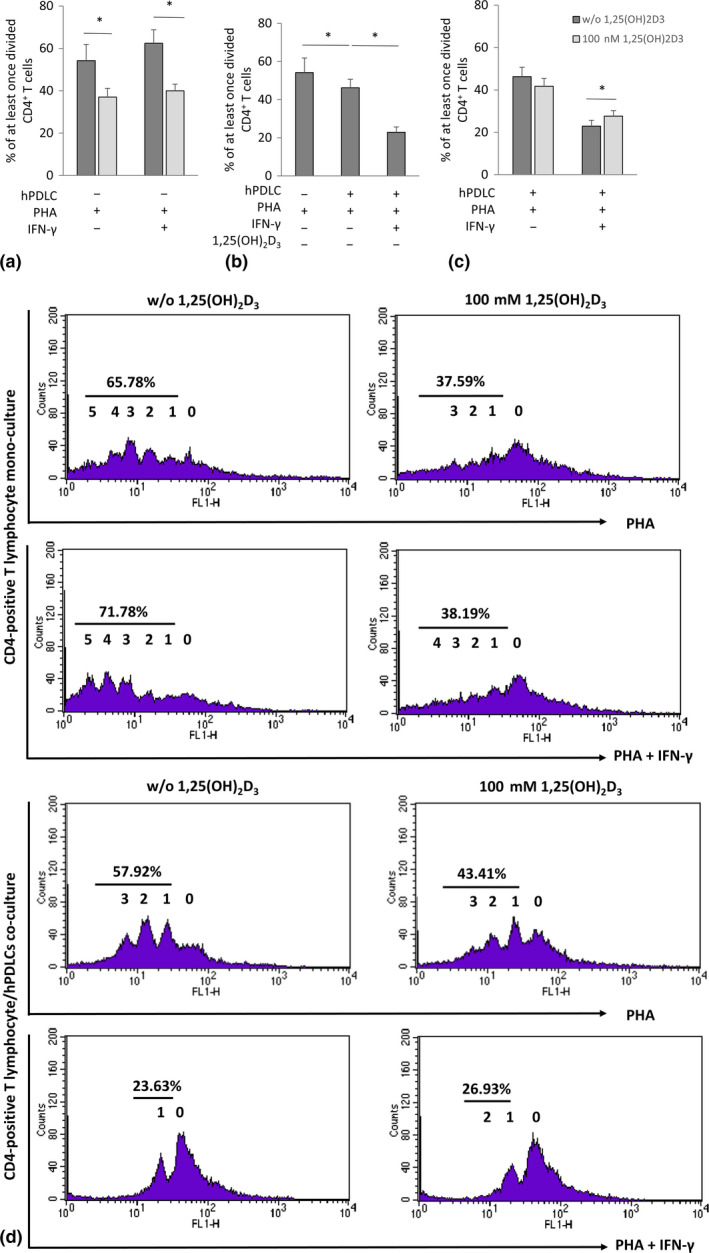
Effect of 1,25(OH)_2_D_3_ on CD4^+^ T lymphocytes proliferation in the presence and absence of hPDLCs. Allogenic CD4^+^ T lymphocytes were activated by 10 µg/ml PHA and co‐cultured with 100 ng/ml IFN‐γ and 100 nM 1,25(OH)_2_D_3_ stimulated hPDLCs for 5 days in an indirect co‐culture model (b, c). PHA‐activated CD4^+^ T lymphocytes, stimulated with different stimuli in the absence of hPDLCs, served as control (A). T‐lymphocyte proliferation was assessed by determining the percentage of at least once divided CFSE‐labelled CD4^+^ T lymphocytes using flow cytometry. (a‐c) show data as mean value ± *SEM* from five independent experiments with hPDLCs isolated from five different individuals. *p‐value < .05 compared between appropriate groups as indicated. (d) shows representative data of one CD4^+^ T‐lymphocyte proliferation assay experiment presented in a one‐parameter histogram. The percentage of at least once divided CD4^+^ T lymphocytes is given. 0 presents the parental generation. 1, 2, 3, 4, 5 and 6 present the first, second, third, fourth, fifth and sixth generation, respectively

Figure 1a‐d shows the effect of 1,25(OH)_2_D_3_ on the PHA‐induced CD4^+^ T‐lymphocyte proliferation in the presence and absence of hPDLCs and/or IFN‐γ. In the absence of hPDLCs, 1,25(OH)_2_D_3_ significantly reduced CD4^+^ T‐lymphocyte proliferation independently from the presence of IFN‐γ (Figure 1a). The proliferation of CD4^+^ T lymphocytes was significantly inhibited by co‐culture with hPDLCs and this effect was enhanced by IFN‐γ (Figure 1b). Under these conditions, 1,25(OH)_2_D_3_ had no significant effect on CD4^+^ T‐lymphocyte proliferation in the absence of IFN‐γ and significantly increased CD4^+^ T‐lymphocyte proliferation in the presence of IFN‐γ (Figure 1c).

### 3.3 CD4^+^ CD25^+^ FoxP3^+^ T_regs_

**Figure 2:**
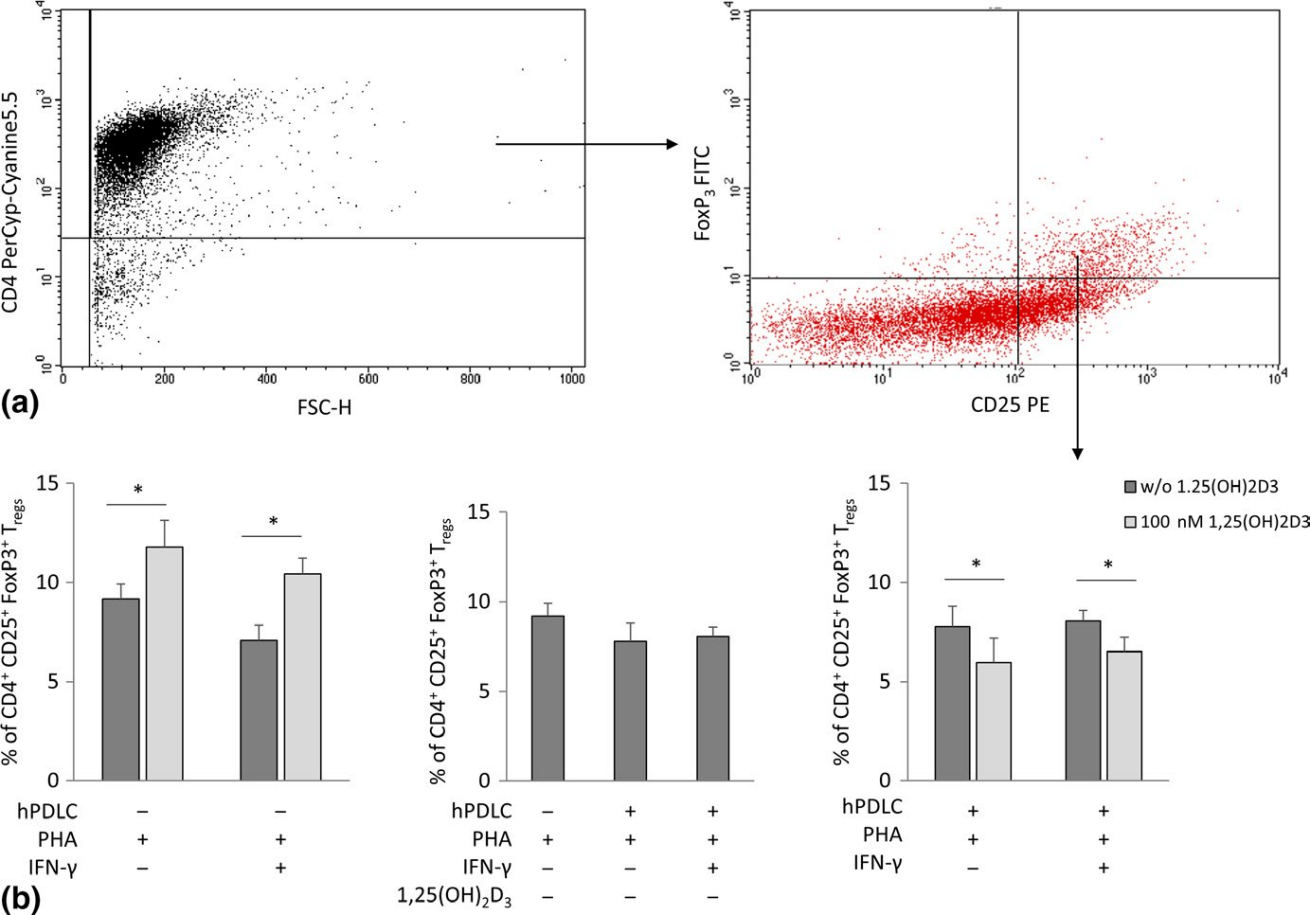
Effect of 1,25(OH)_2_D_3_ on the percentage of CD4^+^ CD25^+^ FoxP3^+^ T_regs_ in the presence and absence of hPDLCs. Allogenic CD4^+^ T lymphocytes were activated by 10 µg/ml PHA and co‐cultured with 100 ng/ml IFN‐γ and 100 nM 1,25(OH)_2_D_3_ treated hPDLCs for 5 days in an indirect co‐culture model. PHA‐activated CD4^+^ T lymphocytes stimulated with different stimuli in the absence of hPDLCs served as control. CD4, CD25 and FoxP3 expression was estimated by immunostaining, followed by flow cytometry analysis. Representative dot plots show the gating strategy of flow cytometry analysis. After gating CD4^+^ T lymphocytes, FoxP3/CD25 double‐positive T lymphocytes were determined (a). Subsequently, the percentage of CD4^+^ CD25^+^ FoxP3^+^ T_regs_ were determined and presented as mean value ± *SEM* from five independent experiments with cells isolated from 5 different individuals (b). *p‐value < .05 compared between appropriate groups as indicated

The effect of 1,25(OH)_2_D_3_ on the proportion of CD4^+^ CD25^+^ FoxP3^+^ T_regs_, evaluated by immunostaining is shown in Figure 2. In the absence of hPDLCs, 1,25(OH)_2_D_3_ increased the percentage of CD4^+^ CD25^+^ FoxP3^+^ T_regs_, independently from the presence of IFN‐γ. Co‐culture of hPDLCs with CD4^+^ T lymphocytes caused a decrease in the percentage of CD4^+^ CD25^+^ FoxP3^+^ T_regs_. Under co‐culture conditions, 1,25(OH)_2_D_3_ significantly decreased the percentage of CD4^+^ CD25^+^ FoxP3^+^ T_regs_ in the absence and in the presence of IFN‐γ.

### 3.4 Production of functional cytokines by CD4^+^ T lymphocytes

**Figure 3:**
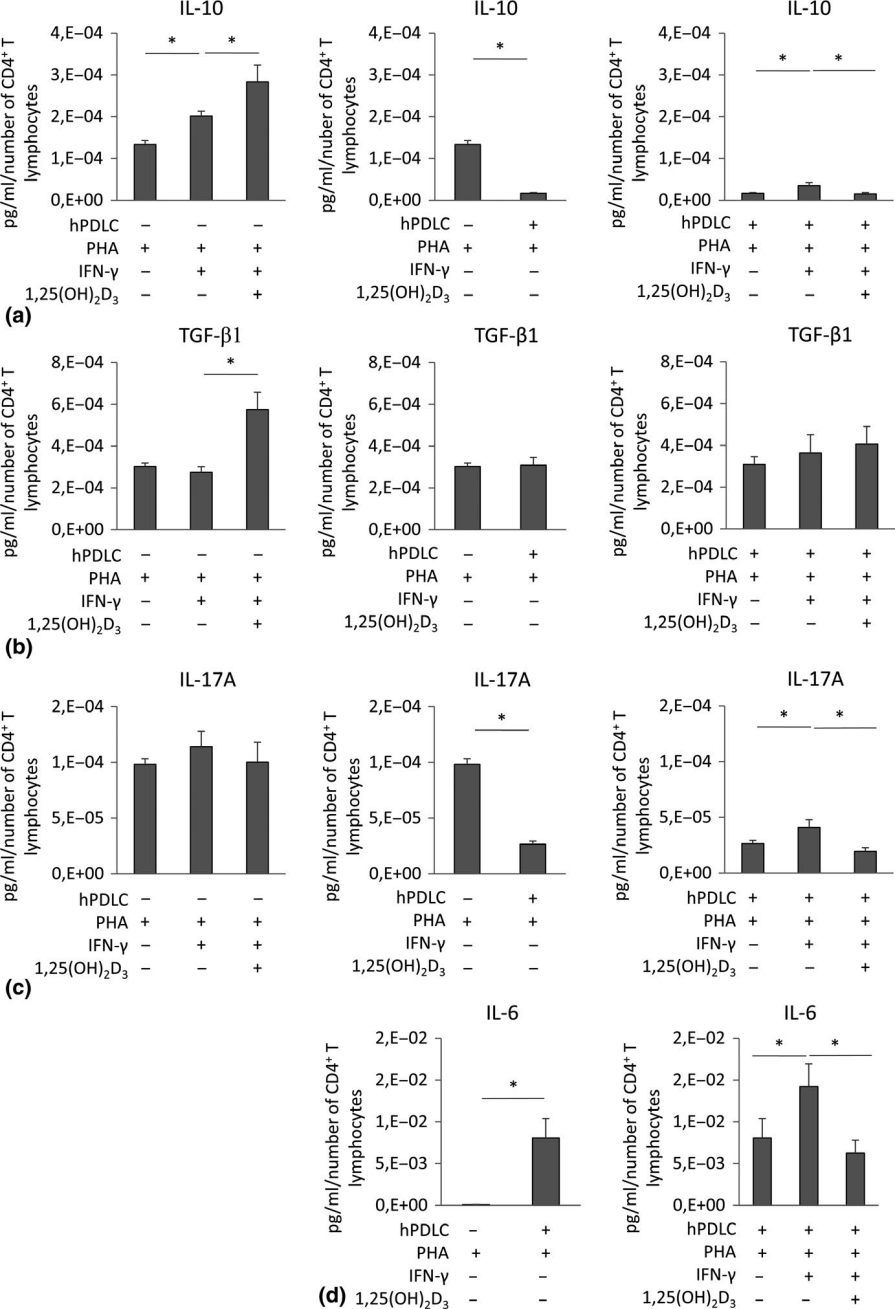
Effect of 1,25(OH)_2_D_3_ on the production of IL‐10, TGF‐β1, IL‐17A and IL‐6 in CD4^+^ T lymphocytes depending on the presence and absence of hPDLCs. Allogenic CD4^+^ T lymphocytes were activated by 10 µg/ml PHA and co‐cultured with 100 ng/ml IFN‐γ and 100 nM 1,25(OH)_2_D_3_ stimulated hPDLCs for 5 days in an indirect co‐culture model. IL‐10 (a), TGF‐β1 (b), IL‐17A (c) and IL‐6 (d) levels were determined in conditioned media using ELISA. Measured cytokine concentrations were normalized to the appropriate total number of CD4‐positive T lymphocytes. All data are presented as mean ± *SEM*. from six independent experiments using hPDLCs isolated from six different individuals. *p‐value < .05 compared between corresponding groups as indicated

The effect of 1,25(OH)_2_D_3_ on the production of the functional cytokines IL‐10, TGF‐β1, IL‐17A and IL‐6 in different experimental settings is shown in Figure 3. In the absence of hPDLCs, 1,25(OH)_2_D_3_ significantly enhanced the expression of IL‐10 (Figure 3a) and TGF‐β1 (Figure 3b), but did not affect the expression of IL‐17A (Figure 3c). In the absence of hPDLCs, PHA‐activated CD4^+^ T lymphocytes produced IL‐6 at hardly detectable levels (Figure 3d) and therefore, the assessment of IFN‐γ‐ or 1,25(OH)_2_D_3_‐induced effect was not possible. Co‐culture of hPDLCs with CD4^+^ T lymphocytes significantly reduced IL‐10 expression (Figure 3a), which was partially recovered in the presence of IFN‐γ. Under this condition, 1,25(OH)_2_D_3_ induced a significant decrease in IL‐10 production. TGF‐β1 expression (Figure 3b) seems to be unaffected by hPDLCs and IFN‐γ. In contrast to monoculture, 1,25(OH)_2_D_3_ did not affect TGF‐β1 production under these conditions. Co‐culture of hPDLCs with CD4^+^ T lymphocytes significantly reduced IL‐17A expression (Figure 3c), but significantly increased IL‐6 (Figure 3d). IFN‐γ treatment of hPDLCs significantly increased IL‐17A and IL‐6 expression, which was significantly diminished in the presence of 1,25(OH)_2_D_3_.

### 3.5 IDO‐1 expression

**Figure 4:**
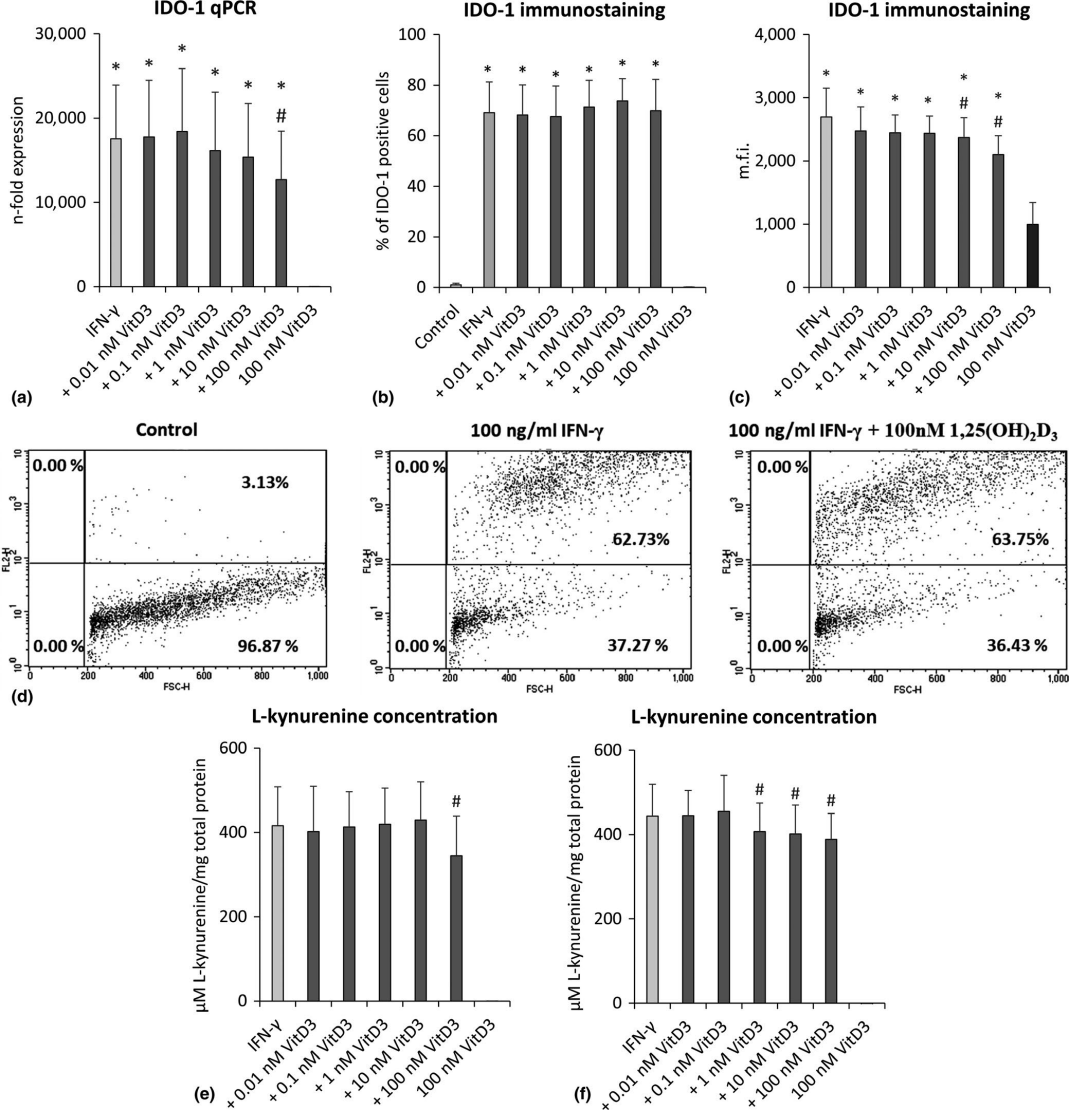
Effect of different 1,25(OH)_2_D_3_ concentrations on IDO‐1 expression and activity in IFN‐γ treated hPDLCs. Primary hPDLCs were stimulated with different 1,25(OH)_2_D_3_ concentrations (0.01–100 nM) in the presence of 100 ng/ml IFN‐γ for 48 hr. IDO‐1 gene expression was investigated by qPCR (a), showing the *n*‐fold expression of IDO‐1 compared to the control (=1). GAPDH served as internal control. Intra‐cellular IDO‐1 protein expression was investigated by intra‐cellular immunostaining and flow cytometry analysis followed by determining the % of IDO‐1 positive hPDLCs (b) and the m.f.i of IDO‐1 positive hPDLCs (c). Representative dot plots which show the percentage of IDO‐1 positive hPDLCs (upper right quadrant) are shown. Quadrants were set using unlabelled control (d). L‐kynurenine concentrations in µM normalized to total protein amount in mg were determined in cell lysates (e) and in the conditioned media (f). Normalized L‐kynurenine concentration of the control was subtracted from each sample. All data are presented as mean value ± *SEM* from five independent experiments with cells isolated from five different individuals. *p‐value < .05 compared to the control; #*p*‐value < .05 compared to IFN‐γ alone

Effect of different 1,25(OH)_2_D_3_ concentrations on the IFN‐γ‐induced IDO‐1 expression and enzymatic activity in hPDLCs are shown in Figure 4. IFN‐γ led to a significant increase in IDO‐1 gene expression in hPDLCs, which was suppressed by 1,25(OH)_2_D_3_ in a dose‐dependent manner (Figure 4a). A significant reduction in IDO‐1 gene expression was observed at 100 nM 1,25(OH)_2_D_3_. Analysis of IDO‐1 protein expression showed that IFN‐γ alone induced a significantly higher percentage of IDO‐1 positive cells. 1,25(OH)_2_D_3_ had no significant effect on the percentage of IDO‐1 positive hPDLCs treated with IFN‐γ (Figure 4b,d), but induced a dose‐dependent decrease in their mean fluorescence intensity (m.f.i., Figure 4c). A statistically significant effect was observed starting from 10 nM 1,25(OH)_2_D_3_. Additionally, IFN‐γ induced an increase in the production of L‐kynurenine in conditioned media (Figure 4f) and cell lysates (Figure 4e). IFN‐γ‐induced IDO‐1 enzymatic activity was suppressed by 1,25(OH)_2_D_3_ in a dose‐dependent manner. In the absence of IFN‐γ, 1,25(OH)_2_D_3_ had no significant effect on IDO‐1 expression and enzymatic activity.

### 3.6 PD‐L1 and PD‐L2 expression

**Figure 5:**
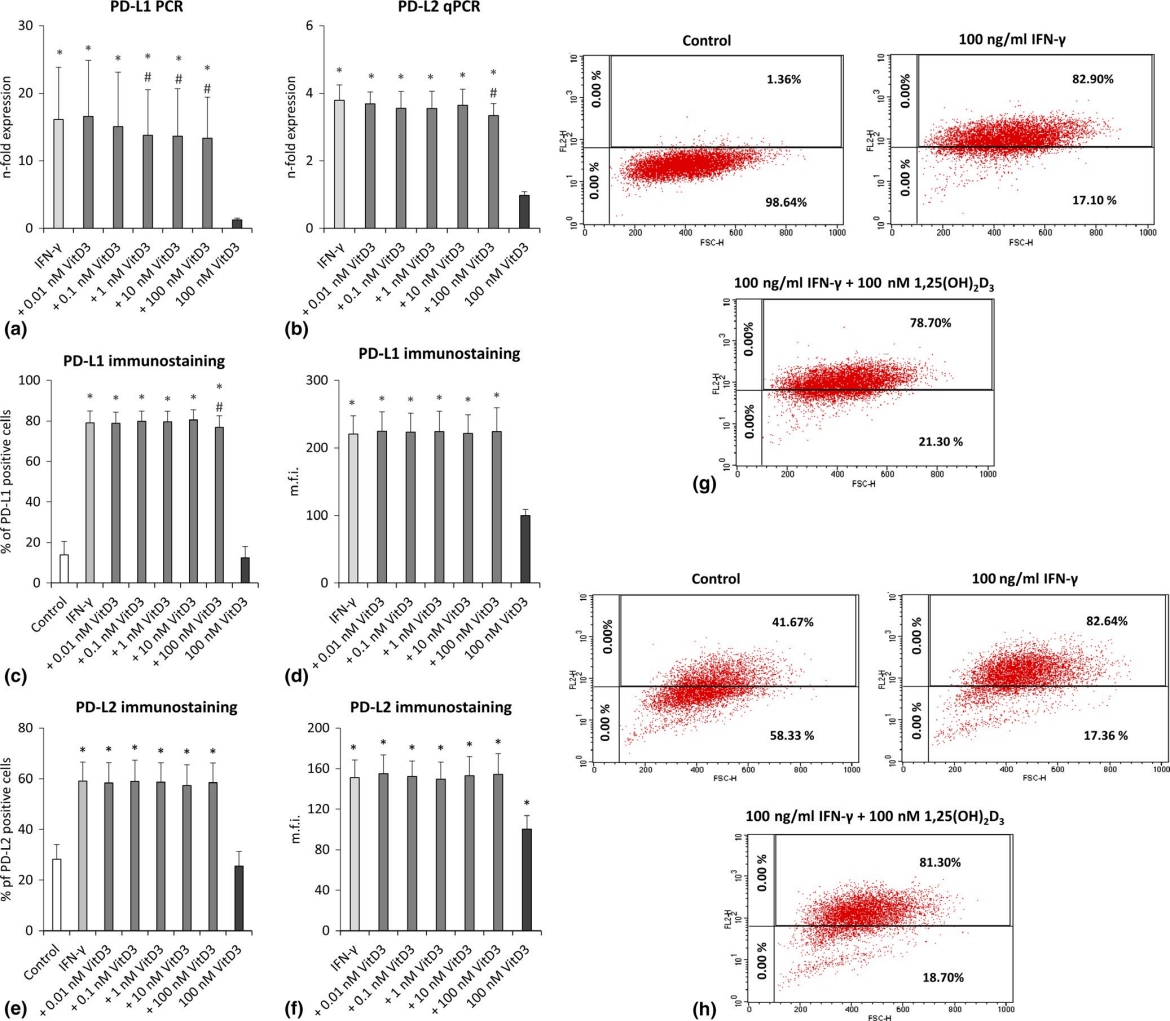
Effect of different 1,25(OH)_2_D_3_ concentrations on PD‐L1 and PD‐L2 expression in IFN‐γ treated hPDLCs. Primary hPDLCs were stimulated with different 1,25(OH)_2_D_3_ concentrations (0.01–100 nM) in the presence of 100 ng/ml IFN‐γ for 48 hr. PD‐L1 (a) and PD‐L2 (b) expression were investigated by qPCR, showing the *n*‐fold expression compared to the appropriate controls. GAPDH served as internal control. PD‐L1 and PD‐L2 surface protein expression was investigated by immunostaining and flow cytometry analysis followed by determining the % of PD‐L1 (c) and PD‐L2 (e) positive hPDLCs and the m.f.i. of PD‐L1 (d) and PD‐L2 (f) positive hPDLCs. Representative dot plots show the percentage of PD‐L1 (g) or PD‐L2 (h) positive hPDLCs (upper right quadrant). Quadrants were set using unlabelled control. All data are presented as mean value ± *SEM* from five independent experiments with hPDLCs isolated from five different individuals. *p‐value < .05 compared to the control; #*p*‐value < .05 compared to IFN‐γ alone

The influence of different 1,25(OH)_2_D_3_ concentrations on the gene and protein expression of PD‐L1 and PD‐L2 in IFN‐γ‐treated hPDLCs is shown in Figure 5. IFN‐γ caused a significant increase in both PD‐L1 and PD‐L2 gene expression in hPDLCs, which was reduced by 1,25(OH)_2_D_3_ in a dose‐dependent manner (Figure 5a,b). A significant effect was observed starting from 1nM 1,25(OH)_2_D_3_ for PD‐L1 and from 100 nM 1,25(OH)_2_D_3_ for PD‐L2. The percentage of PD‐L1 and PD‐L2 positive hPDLCs (Figure 5c,e,g,h) and corresponding m.f.i (Figure 5d,e) were significantly increased after IFN‐γ treatment. Hundred nanomolar 1,25(OH)_2_D_3_ significantly reduced the percentage of PD‐L1 positive IFN‐γ‐treated hPDLCs but had no effect on the percentage of PD‐L2 positive hPDLCs or the m.f.i of both, PD‐L1 and PD‐L2 positive cells.

### 3.7 PTGS‐2 expression

**Figure 6:**
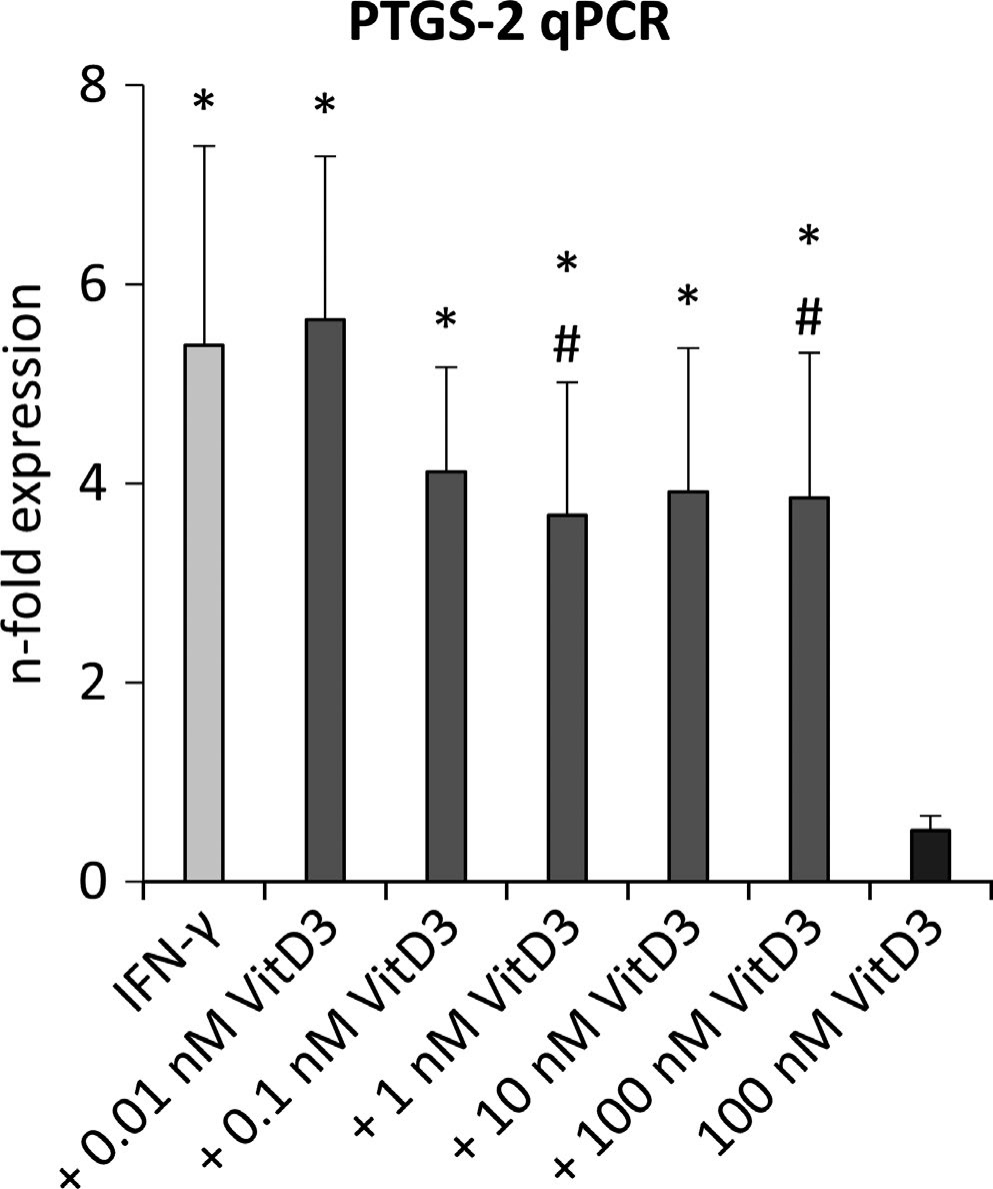
Effect of different 1,25(OH)_2_D_3_ concentrations on PTGS‐2 expression in IFN‐γ treated hPDLCs. Primary hPDLCs were stimulated with different 1,25(OH)_2_D_3_ concentrations (0.01–100 nM) in the presence of 100 ng/ml IFN‐γ for 48 hr. Unstimulated and only with 100 nM 1,25(OH)_2_D_3_ treated cells served as control. PTGS‐2 gene expression was investigated by qPCR, showing the *n*‐fold expression compared to the control. GAPDH served as internal control. All data are presented as mean value ± *SEM* from five independent experiments with hPDLCs isolated from five different individuals. *p‐value < .05 compared to the control; #*p*‐value < .05 compared to IFN‐γ alone

Figure 6 shows the effect of different 1,25(OH)_2_D_3_ concentrations on the PTGS‐2 expression in IFN‐γ treated hPDLCs. IFN‐γ induced a significant increase in PTGS‐2 gene expression, which was inhibited by 1,25(OH)_2_D_3_ in a dose‐dependent manner. Statistically significant effects were observed at 1 and 100 nM 1,25(OH)_2_D_3_. No significant effect of 1,25(OH)_2_D_3_ on the basal PTGS‐2 gene expression was observed in hPDLCs.

### 3.8 IDO‐1, PD‐L1 and PTGS‐2 inhibition

**Figure 7:**
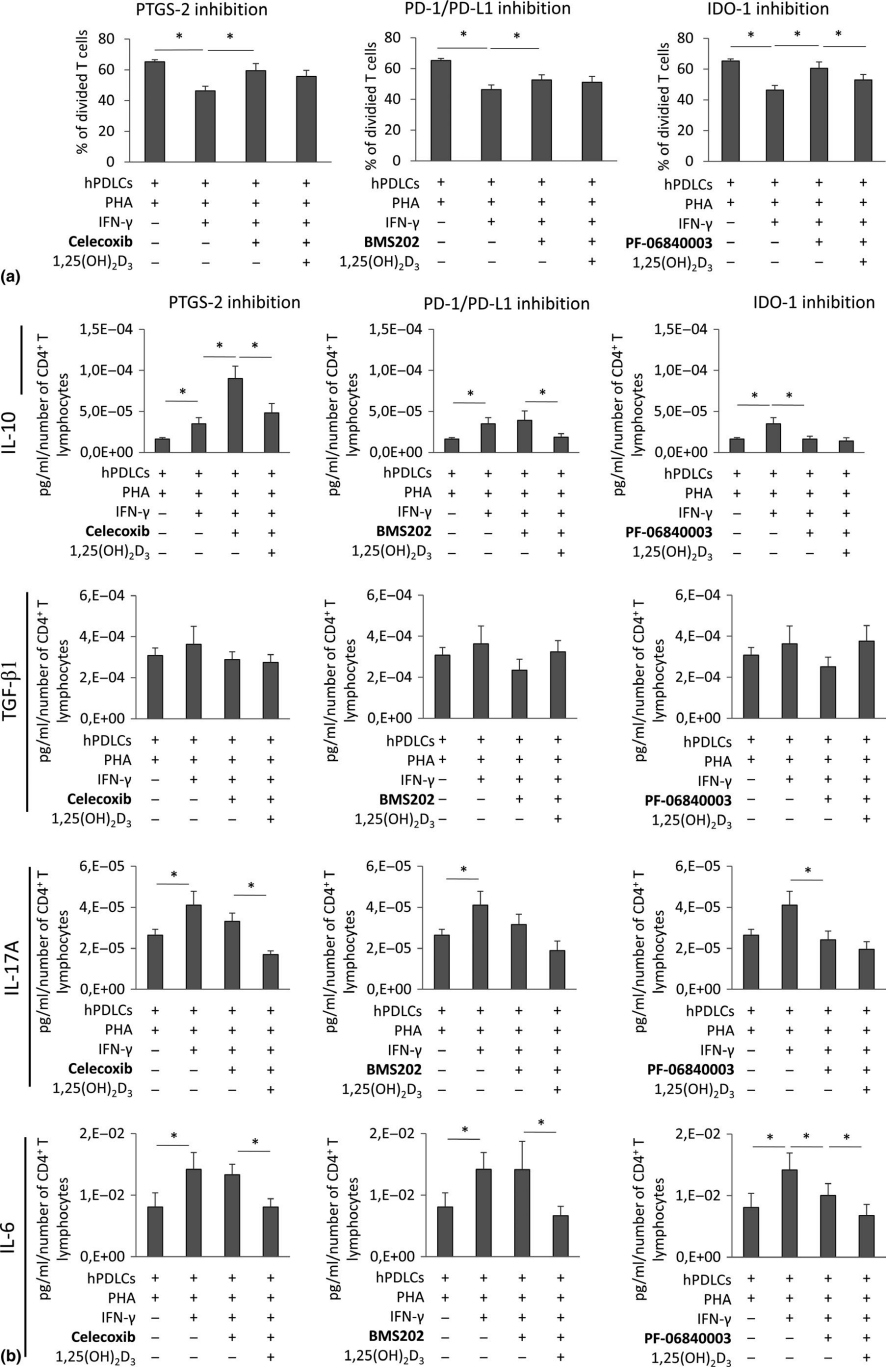
Effect of IDO‐1, PD‐L1 or PTGS‐2 inhibitors on the proliferation and the production of IL‐10, TGF‐β1, IL‐17A and IL‐6 in CD4^+^ T lymphocytes in the presence of IFN‐γ and 1,25(OH)_2_D_3_ treated hPDLCs. Allogenic CD4^+^ T lymphocytes were activated by 10 µg/ml PHA and co‐cultured with IFN‐γ and 1,25(OH)_2_D_3_ stimulated hPDLCs for 5 days in an indirect co‐culture model. Additionally, either 50 µM IDO‐1 inhibitor PF‐06840003 or 1 µM PD‐1/PD‐L1 interaction inhibitor BMS202 or 1 µM PTGS‐2 inhibitor Celecoxib were added to appropriate hPDLCs before and during indirect co‐culture. CD4^+^ T‐lymphocyte proliferation was verified by determining the percentage of at least once divided CFSE‐labelled CD4^+^ T lymphocytes by flow cytometry (a). Additionally, IL‐10, TGF‐β1, IL‐17A and IL‐6 protein levels in conditioned media were determined by appropriate ELISA (b). All data are presented as mean value ± *SEM* from five independent experiments with hPDLCs isolated from five different individuals. * *p*‐value < .05 compared between groups as indicated

Figure 7a shows the role of IDO‐1, PD‐L1 and PTGS‐2 production by hPDLCs on the effect of 1,25(OH)_2_D_3_ on PHA‐induced CD4^+^ T‐lymphocyte proliferation. Pharmacological inhibition of either IDO‐1, PD‐L1 or PTGS‐2 counteracted hPDLCs‐induced suppression of CD4^+^ T‐lymphocyte proliferation by different degrees. In the presence of IDO‐1 inhibitor, a significant reduction of CD4^+^ T‐lymphocyte proliferation by 1,25(OH)_2_D_3_ was observed. In the presence of PD‐L1 and PTGS‐2 inhibitors, 1,25(OH)_2_D_3_ reduced CD4^+^ T‐lymphocyte proliferation, however, without any significance.

Figure 7b shows the production of functional cytokines in the co‐culture experiments in the presence of IDO‐1, PD‐L1 and PTGS‐2 inhibitors. In the absence of 1,25(OH)_2_D_3_, PTGS‐2 inhibition caused a significant increase in IL‐10 production. IDO‐1 inhibition caused a significant decrease in IL‐10, IL‐17A and IL‐6 production. The addition of 1,25(OH)_2_D_3_ caused a significant decrease in the production of all three cytokines in most cases, which was observed even in the presence of inhibitors. Applied inhibitors showed no significant influence on TGF‐β1 protein expression.

## 4 DISCUSSION

In the present study, we used a co‐culture model of PHA‐activated CD4^+^ T lymphocytes and IFN‐γ‐treated hPDLCs in the presence of 1,25(OH)_2_D_3_. IFN‐γ is produced by Th1 lymphocytes and natural killer cells, two important immune cells during periodontitis pathogenesis and is a potent activator of immunomodulatory properties in hPDLCs (Wada et al., 2013). The chosen concentration of 100 ng/ml IFN‐γ is comparable to those in the gingival crevicular fluid of periodontitis patients (Dutzan et al., 2009). Vitamin D_3_ was added to investigate its effect on the interaction between CD4^+^ T lymphocytes and hPDLCs. Several studies showed associations between vitamin D_3_ deficiency (Laky et al., 2017) or vitamin D_3_ receptor polymorphism (Wan, Li, Yang, Liu, & Song, 2019) and periodontitis. Thus, the chosen conditions reflect a clinically relevant situation.

We found that in an indirect co‐culture model, hPDLCs inhibited PHA‐induced proliferation of CD4^+^ T lymphocytes, which is in accordance with previous studies (Liu, Meng, et al., 2012; Wada, Menicanin, Shi, Bartold, & Gronthos, 2009). Furthermore, we showed that the addition of IFN‐γ into the co‐culture results in further inhibition of CD4^+^ T‐lymphocyte proliferation. Since IFN‐γ did not affect CD4^+^ T‐lymphocyte proliferation in the absence of hPDLCs, it can be concluded that this additional inhibition is due to enhancing hPDLCs immunosuppressive properties by IFN‐γ. This enhancement is achieved through up‐regulation of immunomodulatory factors, such as IDO‐1, PD‐L1, PD‐L2 and PTGS‐2, which is in agreement with another study (Wada et al., 2009).

The effect of vitamin D_3_ on the proliferation of CD4^+^ T lymphocytes was strikingly dependent on the experimental conditions. In the absence of hPDLCs and IFN‐γ, vitamin D_3_ significantly inhibited CD4^+^ T‐lymphocyte proliferation, as reported previously (Sheikh et al., 2018). However, this inhibitory effect was not observed under complex co‐culture conditions with hPDLCs. In the presence of hPDLCs, no significant effect of vitamin D_3_ on the proliferation of CD4^+^ T lymphocytes was observed. Thus, although both vitamin D_3_ and hPDLCs separately inhibited CD4^+^ T‐lymphocyte proliferation, their combined application did not result in any additive effect. Most interestingly, vitamin D_3_ induced a statistically significant increase in CD4^+^ T‐lymphocyte proliferation in the presence of IFN‐γ treated hPDLCs. Since the inhibitory effect of vitamin D_3_ on the proliferation of CD4^+^ T lymphocytes was not affected by IFN‐γ in monoculture, the vitamin D_3_ effect in co‐culture is obviously mediated by hPDLCs.

We further investigated the effect of vitamin D_3_ on the proportion of CD4^+^ CD25^+^ FoxP3^+^ T_regs_ under different experimental conditions. The expression of these surface markers is necessary but not a sufficient attribute of Tregs. Additionally, we have investigated the expression of several functionally essential cytokines, such as IL‐6, IL‐10, IL‐17A and TGF‐β1. IL‐10 and TGF‐β1 are characteristic cytokines produced by Tregs (Sakaguchi, 2005), whereas IL‐17A is produced by Th17 lymphocytes (Langrish et al., 2005). There is a tight and complex relationship between Tregs and Th17 lymphocytes and an imbalance between these populations is crucial for the inflammatory response in periodontitis (Campbell, Millhouse, Malcolm, & Culshaw, 2016). Besides, IL‐6 and TGF‐β1 are known to regulate differentiation of Th17 lymphocytes (Kimura & Kishimoto, 2010). In the absence of hPDLCs, vitamin D_3_ induced a significant increase in the proportion of CD4^+^ CD25^+^ FoxP3^+^ T_regs_ and the production of IL‐10 and TGF‐β1, which is in agreement with former studies (Gregori et al.., 2001; Penna et al., 2005; Zhou et al., 2017). However, in the presence of hPDLCs and IFN‐γ, qualitatively different effects of 1,25(OH)_2_D_3_ were observed regarding the proportion of CD4^+^ CD25^+^ FoxP3^+^ T_regs_ and the production of IL‐10, IL‐17A and TGF‐β1. Thus, similarly to CD4^+^ T‐lymphocyte proliferation, the effect of 1,25(OH)_2_D_3_ on these parameters was substantially modified by hPDLCs, but this effect was independent of the presence of IFN‐γ.

It seems that there is no single mechanism explaining the variety of 1,25(OH)_2_D_3_ effects on CD4^+^ T lymphocytes proliferation in the presence of hPDLCs compared to monoculture conditions. We have demonstrated the inhibitory effect of 1,25(OH)_2_D_3_ on the IFN‐γ‐induced protein expression of IDO‐1, PD‐L1 and PTGS‐2, which are known to mediate immunosuppressive effects of MSCs (Andrukhov et al., 2019). The relevance of these mechanisms was partially confirmed by pharmacological inhibition of various immunomodulatory proteins. The suppressive effect of hPDLCs on CD4^+^ lymphocyte proliferation was most strongly reversed upon IDO‐1 inhibition and, by a lesser extent, upon PD‐L1 and PTGS‐2 inhibition. In the presence of all inhibitors, 1,25(OH)_2_D_3_ reduced CD4^+^ T‐lymphocyte proliferation, but a significant effect was only observed for IDO‐1 inhibitor. This confirms that 1,25(OH)_2_D_3_ executes its hPDLCs‐mediated effect on CD4^+^ T‐lymphocyte proliferation via IFN‐γ induced expression of immunomediators. Diminishing of IFN‐γ induced IDO‐1 and, by a lesser extent, PD‐L1 and PTGS‐2 protein expression in hPDLCs by 1,25(OH)_2_D_3_ can abrogate their immunosuppressive effect, which results in high CD4^+^ T‐lymphocyte proliferation.

In contrast to CD4^+^ T‐lymphocyte proliferation, the vitamin D_3_ effects on CD4^+^ CD25^+^ FoxP3^+^ T_regs_ formation and cytokine production in co‐culture with hPDLCs are more complex. Inhibition of IL‐10 production by co‐culture with hPDLCs was partially reversed by PTGS‐2 inhibitors, whereas IDO‐1 inhibition caused a decrease in both IL‐10 and IL‐17A production. In other cases, no essential effect of inhibitors on cytokine production was observed. The effect of 1,25(OH)_2_D_3_ on the cytokine production by T lymphocytes under co‐culture conditions was only partially affected by different inhibitors. However, even in the presence of inhibitors, the effects of 1,25(OH)_2_D_3_ on cytokine production was qualitatively different than those in CD4^+^ T‐lymphocyte monoculture. These data suggest that the 1,25(OH)_2_D_3_ effect on cytokine production by CD4^+^ T lymphocytes via hPDLCs may also be mediated by other mechanisms, which still remain to be investigated.

Our data suggest implicitly that the effect of vitamin D_3_ on the immune response strongly depends on the microenvironment. By translating our data to the situation in vivo, we can assume that regulation of immune response by vitamin D_3_ and hPDLCs may be an essential mechanism of local tissue homoeostasis. Under the conditions that the immune response is not inhibited by hPDLCs, vitamin D_3_ has immunosuppressive effects by inhibiting CD4^+^ T‐lymphocyte proliferation and enhancing the CD4^+^ CD25^+^ FoxP3^+^ T_regs_ proportion. Under certain conditions, the CD4^+^ T‐lymphocyte response might be strongly suppressed by IFN‐γ activated hPDLCs. If this occurs, vitamin D_3_ might partially abolish this suppression and enhance CD4^+^ T‐lymphocyte response. Thus, vitamin D_3_ seems to play an important role in fine‐tuning the periodontal tissue homoeostasis and the local inflammatory response either directly through immune cells or via modulating the immunomodulatory potential of hPDLCs.

Periodontitis is a chronic inflammatory disease (Kinane, 2001) caused by a disruption of host‐microbial homoeostasis (Hajishengallis & Lamont, 2014) and driven by a dysregulated immune response (Hasturk & Kantarci, 2015). In contrast to in vitro studies, which suggest the anti‐inflammatory effect of vitamin D_3_ towards different cells of the periodontium (Andrukhov et al., 2014; Hosokawa et al., 2015; Tang et al., 2013), data of clinical reports are not conclusive. A recent systematic review showed that the role of vitamin D_3_ in chronic periodontitis remains controversial (Perić, Cavalier, Toma, & Lasserre, 2018). Several clinical studies already demonstrated reduced vitamin D_3_ serum levels in periodontitis patients and a negative association between vitamin D_3_ serum levels and the severity of periodontal inflammation (Dietrich, Joshipura, Dawson‐Hughes, & Bischoff‐Ferrari, 2004; Dietrich, Nunn, Dawson‐Hughes, & Bischoff‐Ferrari, 2005). However, other studies detected an increased vitamin D_3_ serum level in periodontitis patients and a positive correlation between vitamin D_3_ serum levels and the severity of periodontal diseases (Liu et al., 2009). The complex co‐culture model used in our study shows that the effect of vitamin D_3_ on the immune response depends on the resident tissue cells and the inflammatory environment and might not always be beneficial. However, translating our data into the clinical situation still has some limitations. We have used cells isolated from the periodontal ligament of third molars, which are usually used for hPDLCs isolation, because these teeth are most often extracted in healthy individuals. However, the restricted participation of these teeth in mastication and rather low mechanical load is a certain limitation for the clinical translation of our data. Nevertheless, the exact role of vitamin D_3_ in periodontitis still needs to be clarified using well‐designed clinical trials and complex in vitro models resembling the structure of the periodontium.

In conclusion, this in vitro study shows that vitamin D_3_ differently affects local CD4^+^ T‐lymphocyte response depending on hPDLCs and their activation by IFN‐γ. These immunosuppressive and immunostimulating effects of vitamin D_3_ on the local inflammatory response may contribute to the local immune homoeostasis in the periodontium and to the balance between periodontal tissue destruction and periodontal pathogen elimination during the progression of periodontitis. The exact role of vitamin D_3_ in influencing the local tissue homoeostasis and the local immune response in periodontitis and its potential as a therapeutic agent against periodontitis has to be further clarified.

## CONFLICT OF INTEREST

The authors declare that the research was conducted in the absence of any commercial or financial relationships that could be construed as a potential conflict of interest.

## Supporting information

Supplementary Material

## Data Availability

The raw data supporting the conclusions of this manuscript will be made available by the authors, without undue reservation, to any qualified researcher.
