# Supplementary material for: Pleiotropic effects of vitamin D3 on CD4+ T lymphocytes mediated by human periodontal ligament cells and inflammatory environment
Source: J Clin Periodontol. 2020 Apr 13;47(6):689–701. doi: 10.1111/jcpe.13283 (PMC7318673; doi:10.1111/jcpe.13283)
Supplement: Supplementary file 1 — Supplementary Material [file JCPE-47-689-s001.docx]

**Supporting Information**

# Pleiotropic effects of vitamin D_3_ on CD4^+^ T lymphocytes mediated by human periodontal ligament cells and inflammatory environment

# Running Title: Vitamin D_3_ differently affects CD4^+^ T-lymphocytes

Behm Christian^1^, Blufstein Alice^1^, Gahn Johannes^1^, Kubin Barbara^1^, Moritz Andreas^1^, Rausch-Fan Xiaohui^1^, Andrukhov Oleh^1*^

^1^University Clinic of Dentistry, Division of Conservative Dentistry and Periodontology, Medial University of Vienna, Vienna, Austria

**^*^Corresponding author:**

Andrukhov Oleh

[oleh.andrukhov@meduniwien.ac.at](mailto:oleh.andrukhov@meduniwien.ac.at)

**Supporting Materials and Methods**

*Cell isolation*

Third molars from 5 periodontally healthy individuals, extracted due to orthodontic reasons, were used to isolate primary hPDLCs as described in our previous study (Andrukhov *et al*, 2016). Before the surgical procedure patients were informed and gave their written consent. The study protocol was approved by the Ethics Committee of the Medical University of Vienna. All procedures were performed according to the Good Scientific Practice Guidelines of the Medical University of Vienna and the Declaration of Helsinki. Isolated hPDLCs were cultured under humidified conditions in Dulbecco’s modified Eagle’s medium (DMEM, Sigma-Aldrich, St. Louis, USA), containing 10% fetal bovine serum (FBS, Gibco, Carlsbad, USA) and 1% penicillin and streptomycin (P/S, Gibco, Carlsbad, USA).
Phenotype of hPDLCs was verified by analyzing the expression of mesenchymal and hematopoietic surface markers. Similar to Chan et al., multiparameter flow cytometry analysis (Chan *et al*, 2014) was performed staining single cell suspensions of hPDLCs simultaneously with various antibodies (all from eBioscience, San Diego, USA) against characteristic MSC surface markers: super bright 436-conjugated mouse anti-human CD29 (Cat# 62-0299-42, RRID: [AB_2688202](http://antibodyregistry.org/AB_2688202)), super bright 600-conjugated mouse anti-human CD105 (Cat# 63-1057-42, RRID: [AB_2802435](http://antibodyregistry.org/AB_2802435)), fluorescein isothiocyanate (FITC)-conjugated mouse anti-human CD90 (Cat# 11-0909-42, RRID: [AB_10668828](http://antibodyregistry.org/AB_10668828)) and PerCP-eFluor 710-conjugated mouse anti-human CD73 (Cat# 46-0739-42, RRID: [AB_10670353](http://antibodyregistry.org/AB_10670353)). hPDLC suspensions were further simultaneously stained with antibodies against various hematopoietic surface markers (all from eBioscience, San Diego, USA): APC-conjugated mouse anti-human CD31 (Cat# 17-0319-42, RRID: [AB_10852842](http://antibodyregistry.org/AB_10852842)), Alexa Fluor 700-conjugated mouse anti-human CD45 (Cat# 56-9459-42, RRID: [AB_2574511](http://antibodyregistry.org/AB_2574511)) and APC-eFluor 780-conjugated mouse anti-human CD34 (Cat# 47-0349-42, RRID: [AB_2573956](http://antibodyregistry.org/AB_2573956)). 300,000 cells were resuspended in a 3% bovine serum albumin (BSA, GE Healthcare, Chicago, USA) solution, supplemented with 0.09% sodium azide. 5µl of each antibody were added together with 5µl Super Bright Complete Staining Buffer (Thermo Fisher Scientific, Waltham, USA), reaching a final volume of 50µl. After incubation and washing, cells were resuspended in a 3% BSA solution and analysed. Additionally, hPDLC suspensions were stained using phycoerythrin (PE)-conjugated mouse anti-human CD146 (Cat# 12-1469-41, RRID: AB-11042581) in a single parameter setting. Fluorescence emissions were analysed by flow cytometry using Attune NxT Flow Cytometer (Invitrogen, Carlsbad, USA). Violet, blue and red laser were used to excite the fluorochromes at 405, 488 or 637nm, respectively, and Attune NxT software (Invitrogen, Carlsbad, USA) was used for calculation. The percentage of cells with the CD105+/CD29+/CD73+/CD90+/CD31-/CD34-/CD45- full phenotype from the starting cell population was calculated using a quadruple-gating strategy (Chan *et al*, 2014) similar to Chan et al. Additionally, the percentage of CD146+ hPDLCs was assessed in a single parameter setting.

Peripheral blood mononuclear cells (PBMCs) were isolated from human whole blood using Ficoll-Paque density gradient centrifugation. CD4^+^ T-lymphocytes were isolated from PBMCs by MagniSort^TM^ Human CD4^+^ T cell enrichment kit (Invitrogen, Carlsbad, USA).

*Co-culture of hPDLC and CD4^+^ T-lymphocytes*

To measure CD4^+^ T-lymphocyte proliferation, CD4^+^ T-lymphocytes were pre-labelled using CellTrace CFSE Cell Proliferation Kit (ThermoFischer Scientific, Waltham, USA). After five days incubation, CD4^+^ T-lymphocytes were harvested and resuspended in 200µl buffer (3% BSA and 0.09% sodium azid in 1xPBS). CD4^+^ T cell proliferation was measured using FACSCalibur Flow Cytometer (Becton Dickinson, Franklin Lakes, USA) equipped with an argon laser (488nm). In total, 10.000 cells were counted per sample and the percentage of at least once divided CD4^+^ T-lymphocytes was determined.

CD4, CD25 and FoxP3 expression was analysed by surface and intracellular immunostaining. CD4^+^ T-lymphocytes were harvested after five days incubation, washed in FACS buffer and stained with PerCyp Cyanine 5.5-conjugated mouse anti-human CD4, clone RPA-T4 (Thermo Fischer Scientific Cat# 45-0049-42, RRID: AB_1518744) and PE-conjugated mouse anti-human CD25, clone BC96 (Thermo Fischer Scientific Cat# 12-0259-42, RRID:AB_1659682) in 50µl FACS buffer for 20 minutes at room temperature in the dark. After surface staining, cells were washed once with FACS buffer and stained intracellularly with FITC-conjugated mouse anti-human FoxP3 antibody, clone 236A/E7 (Thermo Fischer Scientific, Cat# 11-4777-42, RRID:AB_11149498) using FoxP3/Transcription Factor Staining Buffer Set (ebioscience, Waltham, USA). Stained CD4^+^ T-lymphocytes were analysed using FACSCalibur Flow Cytometer. Cell count was limited to 10.000 events. The percentage of CD4^+^ CD25^+^ FoxP3^+^ T_regs_ was determined by gating CD4^+^ T lymphocytes followed by identifying CD25/FoxP3 double-positive T lymphocytes.

The production of characteristic functional cytokines of CD4^+^ T lymphocytes was evaluated by measuring IL-10, TGF-β1, IL-17A and IL-6 cytokine levels in conditioned media after five days incubation. They were measured using IL-10 Human Uncoated enzyme-linked immunosorbent assay (ELISA) Kit (detection range: 300-2pg/ml), IL-6 Human Uncoated ELISA Kit (detection range: 200-2pg/ml) and IL-17A Human ELISA Kit (detection range: 100-1.6pg/ml) (all from Thermo Fischer Scientific, Waltham, USA). TGF-β1 was determined using Human TGF-beta 1 DuoSet ELISA (detection range: 2000-31.3pg/ml, R&D Systems, Minneapolis, USA). Measured concentrations of all cytokines were normalized to the appropriate total number of CD4^+^ T lymphocytes which were counted independently by two investigators using Neubauer Improved C-Chip™ Disposable Counting Chamber.

*Expression of immunomodulatory proteins*

mRNA isolation, reverse transcription to cDNA and qPCR were performed using TaqMan Gene Expression Cells-to-Ct Kit (Applied Biosystems, Foster City, USA) as described in our previous study (Behm *et al*, 2019). qPCR was conducted on an ABI StepOnePlus device (Applied Biosystems, Foster City, USA) using the following instrumental settings: 1x 95°C for 10 minutes followed by 50x 15 seconds at 95°C and 1 minute at 60°C. Target genes were amplified by the following TaqMan Gene Expression Assays (Applied Biosystems, Foster City, USA): IDO-1, Hs00984148_m1; PD-L1, Hs00204257_m1; PD-L2, Hs00228839_m1; PTGS-2, Hs00153133_m1 and GAPDH, Hs99999905_m1. GAPDH served as endogenous reference. All qPCR reactions were performed in duplicates. The point at which the PCR product was first detected above a fixed threshold (cycle threshold, C_t_) was determined for each sample. Changes in the expression of each target gene compared to the control were calculated using the 2^-∆∆Ct^ method:

$$\Delta\Delta C_{t}=\left( C_{t}^{target}-C_{t}^{GAPDH} \right)sample-\left( C_{t}^{target}-C_{t}^{GAPDH} \right)control$$

Intracellular IDO-1 protein expression analysis was performed similarly as described above. hPDLCs were fixed and permeabilized using Intracellular Fixation and Permeabilization Buffer Set (ebioscience, Waltham, USA) following staining with PE-conjugated mouse anti-human IDO-1 antibody, clone eyedio (Thermo Fischer Scientific Cat# 12-9477-42, RRID:AB_2572712). Flow cytometry analysis was performed by FACSCalibur Flow Cytometry. 10.000 events were counted per group. Intracellular IDO-1 protein expression was quantified by determining the percentage of IDO-1 positive cells and the corresponding mean fluorescent intensity (m.f.i.).

For surface expression analysis of PD-L1 and PD-L2, hPDLCs were stained with PE-conjugated mouse anti-human PD-L1 antibody, clone B7-H1 (Thermo Fischer Scientific, Cat# 12-5983-42, RRID:AB_11042286) or PE-conjugated mouse anti-human PD-L2 antibody, clone B7-DC (Thermo Fischer Scientific, Cat# 12-5888-42, RRID:AB_10853342), respectively. Cells were analysed by FACSCalibur Flow Cytometer. In total, 10.000 events were counted per group and the percentage of PD-L1 or PD-L2 positive cells and the corresponding m.f.i. were determined.

L-kynurenine concentration was measured in the conditioned media after stimulation and in the cell lysates after incubating hPDLCs for 3 hours in 1xPBS containing 800µM L-tryptophan (Sigma-Aldrich, St. Louis, USA). Harvested conditioned media and cell lysates were mixed with 30% trichloroacetic acid (Sigma-Aldrich, St. Louis, USA) in 1:3 ratio (v/v) followed by 30 minutes incubation at 65°C. After centrifugation, 125µl sample was mixed with 125µl Ehrlich’s Reagent (0.8% P-dimethylbenzaldehyde in glacial acetic acid, Sigma-Aldrich, St. Louis, USA). After 10 minutes incubation at room temperature, the optical density (OD_492_) was measured in duplicates. L-kynurenine concentrations were calculated by plotting measured absorbance against a standard curve of known L-kynurenine (Sigma-Aldrich, St. Louis, USA) concentrations (Grant et al., 2002; Takikawa et al., 1988). The total protein amount of each group was determined by Pierce bicinchoninic acid (BCA) Protein Assay Kit (ThermoFischer Scientific, Waltham, USA). Colorimetric determination of L-kynurenine concentrations was performed and normalized to the appropriate total protein amount in mg for each sample.

*Statistical analysis*

Statistical analysis was performed using SPSS 24.0 (IBM, Amonk, USA, RRID:SCR_002865). Different groups were compared by using the Friedman test, followed by Wilcoxon test for pairwise comparison. P-values < 0.05 were considered to be statistically significant. All data are presented as mean values ± standard error of the mean (S.E.M.) from 5 independent experiments with cells isolated from 5 different individuals.

**Supporting Table 1**

**Supporting Table 1.** Analysis of mesenchymal stem cell and hematopoietic surface marker expression in hPDLCs. Initial cell population was gated for single cells followed by CD105 expression gating. CD105+ cells were further gated on CD31-/CD29+ cells, followed by gating on CD34-/CD73+ and CD45-/CD90+ cells. This strategy enables to assess the percentages of cells with the CD105+/CD29+/CD73+/CD90+/CD31-/CD34-/CD45- full phenotype from the starting cell population. All data are presented as mean percentage ± S.E.M. Data were obtained from five independent experiments using hPDLCs from five different donors.

|  | Subset 1:  FSC/CD105 | Subset 2: CD31/CD29 | Subset 3: CD34/CD73 | Subset 4: CD45/CD90 | % with full phenotype |
| --- | --- | --- | --- | --- | --- |
|  |  |  |  |  |  |
| **CD105+** | **96.2 ± 0.64** | - | - | - | - |
| CD105- | 3.8 ± 0.64 | - | - | - | - |
| **CD31- / CD29+** | - | **98.6 ± 0.39** | - | - | - |
| CD31+ / CD29+ | - | 0.0 ± 0.03 | - | - | - |
| CD31- / CD29- | - | 1.4 ± 0.41 | - | - | - |
|  | - |  |  |  |  |
| CD31+ / CD29- | - | 0.0 ± 0.00 | - | - | - |
| **CD34- / CD73+** | - | - | **97.9 ± 0.62** | - | - |
| CD34+ / CD73+ | - | - | 0.6 ± 0.07 | - | - |
| CD34- / CD73- | - | - | 1.2 ± 0.53 | - | - |
| CD34+ / CD73- | - | - | 0.4 ± 0.10 | - | - |
| **CD45- / CD90+** | - | - | - | **99.7 ± 0.09** | **92.6** |
| CD45+ / CD90+ | - | - | - | 0.3 ± 0.08 | - |
| CD45- / CD90- | - | - | - | 0.0 ± 0.00 | - |
| CD45+ / CD90- | - | - | - | 0.0 ± 0.00 | - |
|  |  |  |  |  |  |

**References**

Andrukhov O, Andrukhova O, Özdemir B, Haririan H, Müller-Kern M, Moritz A & Rausch-Fan X (2016) Soluble CD14 enhances the response of periodontal ligament stem cells to P. gingivalis lipopolysaccharide. *PLoS One* **11:** e0160848

Behm C, Blufstein A, Gahn J, Noroozkhan N, Moritz A, Rausch-Fan X & Andrukhov O (2019) Soluble CD14 Enhances the Response of Periodontal Ligament Stem Cells to Toll-Like Receptor 2 Agonists. *Mediators Inflamm.* **2019:** 8127301

Chan AKC, Heathman TRJ, Coopman K & Hewitt CJ (2014) Multiparameter flow cytometry for the characterisation of extracellular markers on human mesenchymal stem cells. *Biotechnol. Lett.*

Grant RS, Naif H, Thuruthyil SJ, Nasr N, Littlejohn T, Takikawa O & Kapoor V (2002) Induction of Indolamine 2,3-Dioxygenase in Primary Human Macrophages by Human Immunodeficiency Virus Type 1 Is Strain Dependent. *J. Virol.* **74:** 4110–5

Takikawa O, Kuroiwa T, Yamazaki F & Kido R (1988) Mechanism of interferon-γ action. Characterization of indoleamine 2,3-dioxygenase in cultured human cells induced by interferon -γ and evaluation of the enzyme-mediated tryptophan degradation in its anticellular activity. *J. Biol. Chem.* **263:** 2041–8
